# Brain Dose–Volume Thresholds and Survival in Dogs With Intracranial Tumours Treated With the 10 × 4 Gy Radiotherapy Schedule: A Combined Analysis of Two Trials

**DOI:** 10.1111/vco.70038

**Published:** 2025-12-19

**Authors:** Sergejs Unterkirhers, Valeria Sabina Meier, Carla Rohrer Bley

**Affiliations:** ^1^ Clinic for Radiation Oncology & Medical Oncology, Small Animal Department, Vetsuisse Faculty University of Zurich Zurich Switzerland; ^2^ Department of Physics University of Zurich Zurich Switzerland; ^3^ Radiotherapy Hirslanden Aarau Switzerland

**Keywords:** brain, canine, radiotherapy, tolerance

## Abstract

Dogs with intracranial tumours routinely receive radiotherapy, yet species‐specific dose–volume constraints for normal brain tissue remain undefined. In human radiation oncology, exceeding certain brain dose–volume thresholds markedly increases the risk of radiation‐induced injury (e.g., radionecrosis). Current veterinary practice often extrapolates human guidelines without validation in discrete species, creating a gap in evidence‐based planning. This study aimed to identify brain dose–volume thresholds associated with overall survival (OS) in canine brain‐tumour patients. We pooled data from two prospective randomised trials (*n* = 105 dogs) treated with 10 daily fractions of 4 Gy (total 40 Gy) for intracranial tumours at a single institution. Semi‐automated scripting extracted multiple dose–volume metrics, including generalised equivalent uniform dose (gEUD), for the whole brain and brain minus gross tumour volume (Brain–GTV). An iterative Kaplan–Meier and Cox proportional hazards approach identified optimal dosimetric cutoffs, which were then adjusted for tumour volume and body weight via a regression residual method. A brain‐volume‐adjusted gEUD threshold was also derived to account for variation in brain size. Exposure to normal brain to doses around 30–40 Gy emerged as the strongest predictor of OS. Brain–GTV V32 Gy ≤ 13 cm^3^ was associated with longer OS (covariate‐adjusted cutoff 13.4 cm^3^, HR = 1.74; *p* = 0.022, unadjusted optimal split 11.5 cm^3^, HR = 2.08; *p* = 0.001). Whole‐brain gEUD > 30 Gy similarly predicted poorer survival (HR = 1.72; *p* = 0.034). Implementing a personalised gEUD ceiling increased 2‐year sensitivity from 31% to 38% with only a three‐point drop in specificity. In a 10 × 4 Gy canine intracranial radiotherapy model, limiting Brain–GTV V32 Gy to ≤ 13 cm^3^ and whole‐brain gEUD to ≤ 30 Gy was associated with longer overall survival. A brain‐volume‐adjusted gEUD ceiling further refined risk prediction. These evidence‐based thresholds provide actionable guidance for veterinary treatment planning, with the potential to improve outcomes in canine brain tumour therapy.

## 1 Introduction

Radiation therapy plays a significant role in managing intracranial tumours in dogs. However, optimal treatment planning is essential to minimise the risk of severe brain toxicity. Despite its importance, dose–volume constraints for the normal canine brain remain poorly defined. In veterinary neuro‐oncology, radiotherapy fractionation approaches—span conventional definitive protocols (≈45–50 Gy in 18–20 daily fractions) [1, 2, 3, 4, 5, 6], moderately hypofractionated schedules (e.g., 10 × 4 Gy) [4, 7, 8], and stereotactic regimens (e.g., 3–5 fractions) [9, 10, 11, 12]. Multiple studies report median overall survival ~1 to 2 years following definitive RT for canine intracranial tumours, underscoring the clinical benefit of radiotherapy in this setting. Conventional definitive fractionation (≈45–50 Gy in 18–20 fractions) yields median survivals ~16 to 18 months in contemporary series [2, 3, 4, 5, 6]. Hypofractionated protocols that shorten treatment (e.g., 10 × 4 Gy) show outcomes comparable to 20‐fraction schedules, with no significant difference in survival or tumour control [4, 7, 8, 13]. Similarly, stereotactic RT (e.g., 3 × ~8 Gy or 5 daily fractions) achieves survival almost comparable to conventional fractionation in selected settings (e.g., pituitary masses) while reducing anaesthetic episodes and overall treatment time [9, 10, 11].

Prior studies suggest that fractionation and irradiated volume may influence outcomes [4]. Our group has previously developed normal tissue complication probability (NTCP) models for fractionated protocols, incorporating variations in fraction size and irradiated volume to produce risk profiles for different dose–volume combinations [13]. Nevertheless, these theoretical models have yet to undergo underlying clinical validation. Retrospective data indicate that excessive high‐dose volumes are associated with poorer outcomes. For example, Griffin et al. reported that dogs treated with a stereotactic regimen of 3 × 8 Gy experienced significantly higher rates of neurologic decline and early mortality within 6 months when larger volumes of normal brain received high‐dose exposure [12]. These findings underscore that evidence‐based dose–volume constraints are as critical in veterinary radiotherapy as they are in human oncology. To date, however, veterinary literature lacks prospectively collected, controlled data to define clear tolerance thresholds for the canine brain.

To address this gap, we conducted a pooled analysis of prospective randomised clinical trials, including 105 dogs, forming a robust cohort. The trials encompassed both standard and dose‐escalated protocols, providing a broad basis for analysis. We evaluated a broad array of brain dose–volume metrics—including absolute volumes of brain receiving various dose levels, relative volumes and dose percentiles to determine their association with overall survival (OS). Kaplan–Meier curves and Cox proportional hazards models were applied, using an iterative algorithm to determine optimal cutoff values for each dosimetric parameter, thereby stratifying patients into high‐ and low‐risk groups. All analyses were repeated with adjustment for potential confounders (tumour volume and body weight) to ensure that observed dose–volume effects on survival were independent of tumour burden or intrinsic patient life expectancy. The objective of the study was to derive evidence‐based normal brain dose constraints for canine radiotherapy planning and ultimately improve outcomes for veterinary neuro‐oncology patients.

## 2 Materials and Methods

### 2.1 Patient Cohort and Treatment

Patient data were pooled from two prospective randomised trials, conducted at a single institution, investigating 10 × 4 Gy radiotherapy schedules in dogs with intracranial tumours [7]. All protocols received approval from the Animal Ethics Council of the Canton of Zurich, Switzerland (permits ZH075/17 and ZH021/19), and owners provided informed consent. The combined cohort included 105 canine patients (56 from Study 1 and 49 from Study 2) with intracranial lesions defined as gross tumour volume (GTV), treated with dynamic intensity‐modulated radiotherapy (IMRT) or volumetric arc radiotherapy (VMAT). Tumour diagnoses were imaging‐based on MRI, as histopathologic confirmation was not available in most cases.

All patients were treated with 40 Gy in 10 daily fractions (Monday–Friday). In Study 1, dogs were randomised to receive either a homogeneous dose distribution or heterogeneous ‘dose‐painting’ plan that delivered selective intratumoral dose escalation (up to ~120% of prescription, ~48 Gy) within the target. Study 2 compared a standard regimen (10 fractions of 4.0 Gy, total 40 Gy) to a simultaneous integrated boost (SIB) delivering ~46 Gy (115%) to the GTV. Overall survival (OS) was defined from start of radiotherapy to death from any cause; patients alive at last follow‐up were censored.

### 2.2 Dose–Volume Parameter Extraction

Radiotherapy dose distributions were analysed for each patient to extract a comprehensive set of brain dose–volume metrics using semi‐automated scripts of the Eclipse Treatment Planning System API (Varian a Siemens Healthineers company, Palo Alto, California, USA). Trial data were consolidated into a unified database with standardised metric definitions. Metrics were derived for both the whole brain and the brain minus GTV (Brain–GTV) and included:

- *Absolute volumes (V*_*X*_, *cm* ^*3*^): Volume of the structure receiving ≥ *X* Gy (e.g., Brain *V*_20_), calculated for thresholds from 10 to 50 Gy, in 2–3 Gy increments.
- *Relative volumes (V*_*X*_ *%*): Absolute volume normalised to total structure volume (e.g., Brain *V*_20_%).
- *Dose percentiles (D*_*y*_): Minimum dose received by the *y*% of the volume including *D*_max_, *D*_mean_, *D*_2%_, *D*_33%_, *D*_50%_ (median), *D*_66%_ and *D*_98%_.
- *Generalised equivalent uniform dose (gEUD)*: Computed for both whole brain and Brain–GTV using exponent *a* = 4, based on human radiobiological parameters and prior canine application [13, 14].
- *Brain volume adjusted gEUD*: Personalised gEUD normalised to brain size (46.9–123.7 cm^3^). We converted gEUD into a ‘dose‐integral’ $I={\text{gEUD}}_{\text{phys}}^{a}\cdot V_{\text{brain}}$, where a is gEUD exponent (four for brain as described above), ${\text{gEUD}}_{\text{phys}}$ is the physical (10‐fraction) gEUD, and 𝑉_brain_ the contoured brain volume. We scanned 100 candidate cut‐points across the 10th–90th percentile of 𝐼, using two‐sided log‐rank tests to identify a critical integral (*I*_crit_) that minimised the *p‐*value. This *I*_crit_ was back‐transformed to a personalised gEUD ceiling ${\text{gEUD}}_{\max}\left(V\right)={\left(I_{\text{crit}}/V\right)}^{1/4}\;\mathrm{Gy}$. To benchmark the personalised ceiling against a fixed gEUD threshold, each split served as a binary classifier of mortality at 1, 2 and 3 years. Dogs censored prior to each landmark time (*t*_0_ = 365, 730, 1095 days) were excluded; events were defined as deaths on or before *t*_0_.

### 2.3 Statistical Analysis and Threshold Identification

All statistical analyses were performed in Python using the *lifelines* and *scikit‐survival* libraries. Dogs still alive at the time of data evaluation or lost to follow‐up were censored. Survival distributions were estimated by the Kaplan–Meier analysis, with group comparisons via log‐rank test. Cox proportional hazards models quantified hazard ratios (HRs) and 95% confidence intervals (CIs) for the effect of dose–volume metrics on OS. Proportional hazards assumptions were verified with Schoenfeld residuals and graphical checks, and no significant violations were observed.

#### 2.3.1 Univariate Threshold Optimisation

For each dosimetric metric, candidate thresholds spanning the 10th–90th percentiles were evaluated. Patients were dichotomized at each cutoff, and log‐rank tests assessed survival differences. The threshold yielding the smallest *p*‐value (largest log‐rank statistic) was selected. Kaplan–Meier curves and univariate Cox models were then generated for the optimal split, reporting median survival differences and HRs. Statistical significance was set at *p* < 0.05 (two‐tailed).

#### 2.3.2 Adjustment for Covariates

To adjust for confounders (body weight and tumour volume), we applied a regression residual approach. Each dose–volume variable was regressed on weight and GTV, and the residuals (unexplained variation) were used for thresholds optimisation as follows: The resulting optimal cutoffs on residuals were translated back to the original units at mean weight and GTV, yielding clinically interpretable adjusted thresholds. Associations of these adjusted thresholds with OS were then evaluated via log‐rank tests and Cox models.

#### 2.3.3 Bootstrap and Multiple‐Testing Control

To evaluate the stability of identified cut‐points we applied nonparametric bootstrap resampling (*B* = 1000). For each bootstrap resample we re‐optimised candidate cut‐points on a dense grid spanning each metric's resample‐specific 10th–90th percentiles and recorded the resample‐specific optimal cut‐point and adjusted hazard estimate (covariates: weight, GTV; ridge penalizer = 0.1). We summarise the distribution of bootstrap‐selected cut‐points (median, IQR) and the proportion of resamples with adjusted *p* < 0.05 as measures of stability. To control multiplicity across the prespecified family of correlated high‐dose metrics (Brain–GTV V29, V32, V35, V38 and whole‐brain gEUD) we applied Benjamini–Hochberg (BH) FDR adjustment; as a conservative sensitivity analysis we also report Benjamini–Yekutieli (BY) *q*‐values. Reported *p*‐values for the primary family are provided both unadjusted and FDR/BY‐adjusted.

#### 2.3.4 Sensitivity Analysis by Tumour Location

Tumour location can influence dose distributions in the brain; for example, pituitary tumours versus brainstem tumours, and also pituitary tumours can have inherently better survival than other tumour types. To assess whether the observed association between Brain–GTV dose metrics and overall survival was driven by tumour location, we performed sensitivity analyses excluding pituitary tumours and repeated the primary Kaplan–Meier and log‐rank comparisons for selected most influential dose volume parameters using the same thresholds.

Additional statistical tests included Shapiro–Wilk tests for normality, *t*‐tests for approximately normally distributed variables, and Mann–Whitney U tests for non‐normal data. All tests were two‐sided with *α* = 0.05.

#### 2.3.5 Data Availability Statement

The data that support the findings of this study are available from the corresponding author upon reasonable request.

#### 2.3.6 Cell‐Line Validation Statement

Not applicable. This study did not use cell lines.

## 3 Results

### 3.1 Patient and Tumour Characteristics

Table 1 summarises the baseline patient and tumour characteristics. The median age of the combined cohort was 9.2 years (range 3.2–14.5). Median body weight was 14.4 kg (range 2.0–40.8). The sex distribution was 42% male and 58% female. Brachycephalic breeds were frequent and represented 43% of cases.

**TABLE 1:** Patient and tumour characteristics.

Characteristic	Pooled patients (*N* = 105)
Age, years (median [range])	9.2 [3.2–14.5]
Sex: male, *n* (%)	44 (41.9%)
Sex: female, *n* (%)	61 (58.1%)
Brachycephalic breed, *n* (%)	45 (42.9%)
Non‐brachycephalic breeds, *n* (%)	60 (57.1%)
Weight, kg (median [range])	14.4 [2.0–40.8]
Tumour type: meningioma, *n* (%)	47 (44.8%)
Tumour type: glioma, *n* (%)	38 (36.2%)
Tumour type: pituitary, *n* (%)	20 (19.0%)
Tumour location: forebrain, *n* (%)	64 (61.0%)
Tumour location: pituitary, *n* (%)	20 (19.0%)
Tumour location: cerebellum, *n* (%)	4 (3.8%)
Tumour location: brainstem, *n* (%)	9 (8.6%)
GTV volume, cm^3^ (median [range])	2.8 [0.2–11.8]
PTV volume, cm^3^ (median [range])	11.3 [2.4–44.0]
Brain volume, cm^3^ (median [range])	89.2 [46.9–123.7]
RT protocol control, *n* (%)	52 (49.5%) (Control)
RT protocol test, *n* (%)	53 (50.5%) (Experimental)

Abbreviations: brachycephalic, short‐nosed breed; GTV, gross tumour volume; PTV, planning target volume; SIB, simultaneous integrated boost.

The intracranial tumour types were predominantly presumptive meningiomas (≈45% of dogs), gliomas (~36%) and pituitary tumours (~19%). Tumour locations were mainly forebrain (61% of cases), followed by pituitary region (19%), cerebellum (4%) and brainstem (9%). The median GTV was 2.8 cm^3^ (approximately 4% of brain volume), with a median planning target volume (PTV) of 11.3 cm^3^. The median whole‐brain volume was 89.2 cm^3^. Overall, there were no significant baseline differences between the two trial populations, so data were analysed in aggregate.

### 3.2 Protocol Assignments

Across the combined cohort, approximately half of the dogs (*n* = 52, 49.5%) were treated with a uniform 40 Gy/10‐fraction protocol, while the other half (*n* = 53, 50.5%) received an escalated‐dose protocol (heterogeneous dose‐painting in Study 1 or SIB in Study 2).

### 3.3 Overall Survival Outcomes

**FIGURE 1:**
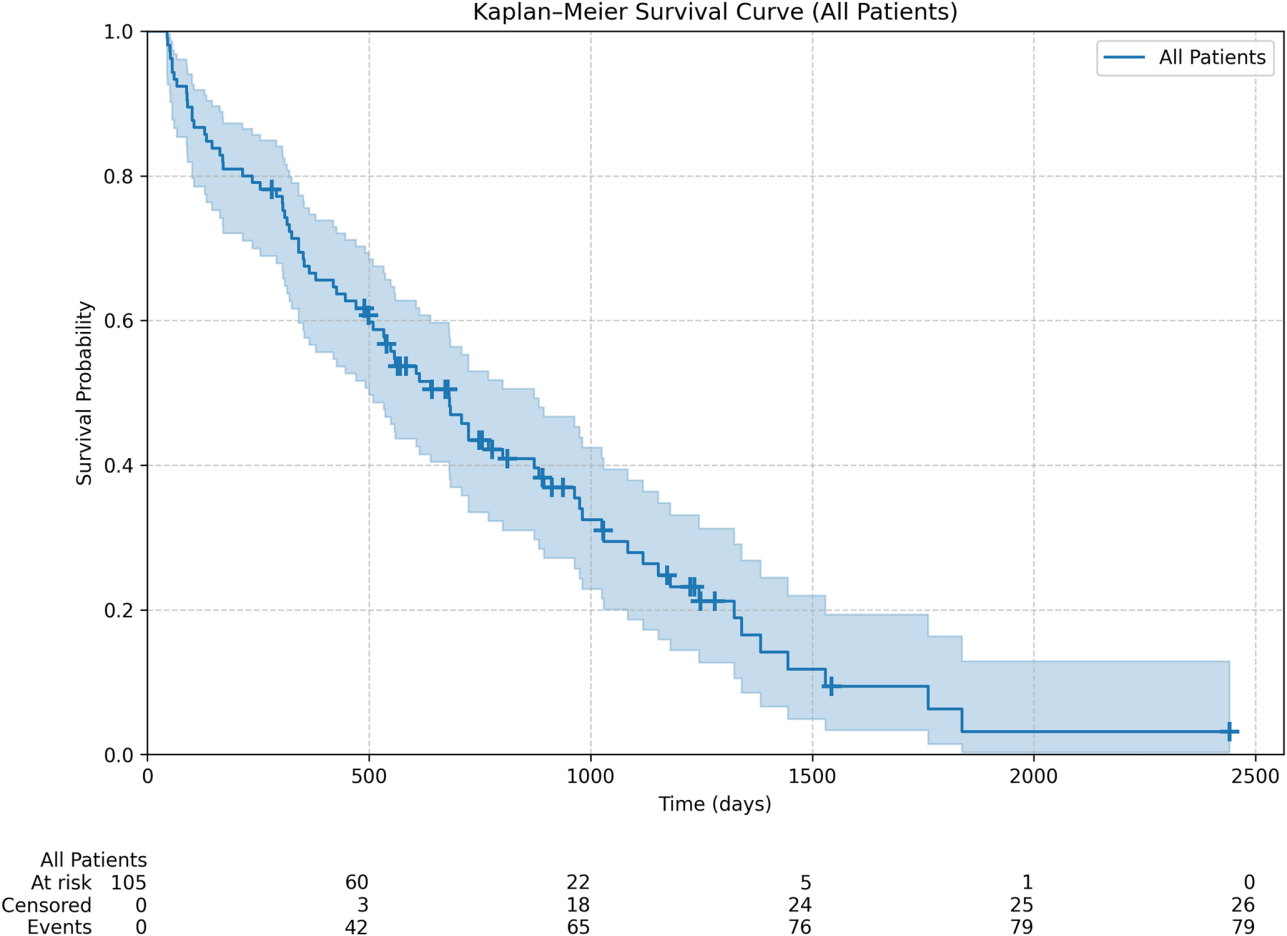
Overall survival probability as function of follow‐up time for combined cohort of patients: the pooled median overall survival (OS) was 681 days (95% CI: 500–895).

The median OS for the combined cohort (*N* = 105) was 681 days (~22.4 months; 95% CI: 500–895 days). One‐year and two‐year survival probabilities were 67% and 43%, respectively. By the end of the observation period, 79 dogs (75%) had died. Overall median follow‐up time was 559 days (range 45–2441 days). For the dogs alive at last follow‐up (censored patients), the median follow‐up time was 728 days (range 281–2441 days) (Figure 1).

### 3.4 Dose–Volume Threshold Analysis (Univariate)

Of the 44 dose–volume metrics tested, those reflecting higher dose exposures (approximately ≥ 30 Gy) in normal brain showed the strongest associations with OS on univariate analysis (Table S1).

**FIGURE 2:**
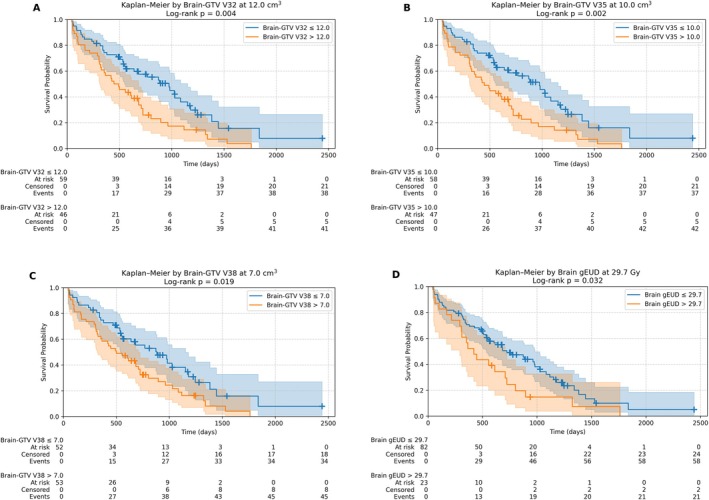
Kaplan–Meier survival curves for selected dose–volume thresholds (Brain–GTV and whole brain). (A) Brain–GTV V32 Gy > 11.5 versus ≤ 11.5 cm^3^. (B) Brain–GTV V35 Gy > 9.6 versus ≤ 9.6 cm^3^. (C) Brain–GTV V38 Gy > 7.3 versus ≤ 7.3 cm^3^. (D) Brain–GTV gEUD > 28 versus ≤ 28 Gy. (All shown thresholds yielded *p* < 0.05; see text for hazard ratios.)

The volume of normal brain (Brain–GTV) receiving > 32 Gy was the single strongest predictor of OS (Figure 2), with an optimal cutoff of 11.5 cm^3^ that stratified dogs into high‐ and low‐risk groups (HR = 2.08, 95% CI: 1.32–3.26, *p* = 0.001). Similar associations were observed at high‐dose levels: Brain–GTV V35 Gy > 9.6 cm^3^ (HR = 2.05, 95% CI: 1.31–3.22, *p* = 0.002), Brain–GTV V38 Gy > 7.3 cm^3^ (HR = 1.81, 95% CI: 1.16–2.83, *p* = 0.009) and Brain–GTV V29 Gy > 13.2 cm^3^ (HR = 1.82, *p* = 0.010). In addition, a Brain–GTV gEUD of > 28 Gy emerged as a significant predictor of OS (HR = 1.79, 95% CI: 1.03–3.10, *p* = 0.038).

Whole‐brain metrics mirrored this pattern. Dogs with a whole‐brain gEUD ≥ 29.7 Gy had significantly reduced OS (HR = 1.72, 95% CI: 1.04–2.86, *p* = 0.034). Higher‐dose whole‐brain volume parameters—specifically Brain V41 Gy > 9.4 cm^3^ (HR = 2.03, 95% CI: 1.17–3.53; *p* = 0.013) and Brain V26 Gy > 21.2 cm^3^ (HR = 1.59, 95% CI: 1.02–2.48; *p* = 0.043)—were also associated with significantly poorer outcomes. Lower‐dose exposures (e.g., V20 Gy) did not reach statistical significance in this cohort.

### 3.5 Covariate‐Adjusted Dose–Volume Analysis

After adjusting for tumour volume and body weight, all three Brain–GTV volume metrics remained significant predictors, with higher cutoff values: V32 Gy > 13.4 cm^3^ (HR = 1.74, 95% CI: 1.10–2.76, *p* = 0.022); V35 Gy > 10.4 cm^3^ (HR = 1.75, 95% CI: 1.12–2.75, *p* = 0.016); V38 Gy > 8.2 cm^3^ (HR = 1.65, 95% CI: 1.05–2.60, *p* = 0.031). Whole‐brain gEUD > 30.1 Gy was also significant (HR = 2.22, 95% CI: 1.18–4.19, *p* = 0.014).

These findings confirm that excessive high‐dose exposure of normal brain tissue remains independently associated with worse overall survival.

### 3.6 Cut‐Off Stability, Effect Sizes and Multiple‐Testing Adjustment

Across 1000 resamples, optimal cut‐offs were stable for the leading predictors. The whole‐brain gEUD had a median bootstrapped cut‐off of 28.50 Gy (IQR: 25.04–29.76 Gy). Brain–GTV V32 showed a median optimal threshold of 11.41 cm^3^ (IQR: 11.22–11.50 cm^3^), close to the single sample estimate. Other top metrics were similarly consistent: V35 9.51 cm^3^ (IQR: 8.67–9.61), V29 13.43 cm^3^ (IQR: 13.23–14.23) and Brain–GTV gEUD 24.05 Gy (IQR: 23.63–27.29). V38 was less stable: median 6.89 cm^3^ with a wider IQR (4.48–7.26).

Bootstrap effect estimates were consistently approximately twofold above the cut‐offs: whole‐brain gEUD median adjusted HR 2.11 (IQR: 1.81–2.51); V32 2.10 (IQR: 1.75–2.40); V35 2.02 (IQR: 1.66–2.33); V29 1.92 (IQR: 1.64–2.26); Brain–GTV gEUD 2.00 (IQR: 1.58–2.43). For V38, the median adjusted HR was 1.87 with a broader IQR (0.61–2.23), indicating greater variability.

Controlling FDR within a pre‐specified family (Brain–GTV V29, V32, V35, V38, whole‐brain gEUD), V32/V35/V38 remained significant by BH and BY (q_BY = 0.008, 0.008, 0.014; adjusted HRs = 2.34, 2.25, 2.01). V29 also remained significant (q_BY = 0.032; HR = 1.83). Whole‐brain gEUD remained significant by BH (q_BH = 0.022; HR = 1.80) and was borderline by BY (q_BY = 0.050). These corrections confirm the robustness of the main associations, particularly V32–V38 (Table 2).

**TABLE 2:** Comparison of selected dose–volume thresholds before and after adjustment for tumour volume and body weight.

Dose–volume metric	Unadjusted threshold	HR (95% CI), unadjusted	*p* (unadjusted)	Adjusted threshold	HR (95% CI), adjusted	*p* (adjusted)
Brain–GTV V32 Gy	11.5 cm^3^	2.08 (1.32–3.26)	0.001	13.36 cm^3^	1.74 (1.10–2.76)	0.019
Brain–GTV V35 Gy	9.6 cm^3^	2.05 (1.31–3.22)	0.002	10.39 cm^3^	1.75 (1.12–2.75)	0.015
Brain–GTV V38 Gy	7.3 cm^3^	1.81 (1.16–2.83)	0.009	8.15 cm^3^	1.65 (1.05–2.60)	0.031
Whole‐brain gEUD	29.7 Gy	1.72 (1.04–2.86)	0.034	30.07 Gy	2.22 (1.18–4.19)	0.01
Brain‐GTV gEUD	27.4 Gy	1.59 (0.95–2.64)	0.077	27.8 Gy	1.79 (1.03–3.10)	0.038
Individualised Whole‐brain gEUD	*I*_crit_ = 6.06e + 07 Gy^4.0^ cm^3^	1.81 (1.11–2.95)	0.017	—	—	—

Abbreviations: Brain–GTV, brain minus gross tumour volume; gEUD, generalised equivalent uniform dose; *I*_crit_, critical dose–volume integral threshold (Gy^4^ cm^3^).

### 3.7 Patient Brain Volume Specific gEUD Analysis

**FIGURE 3:**
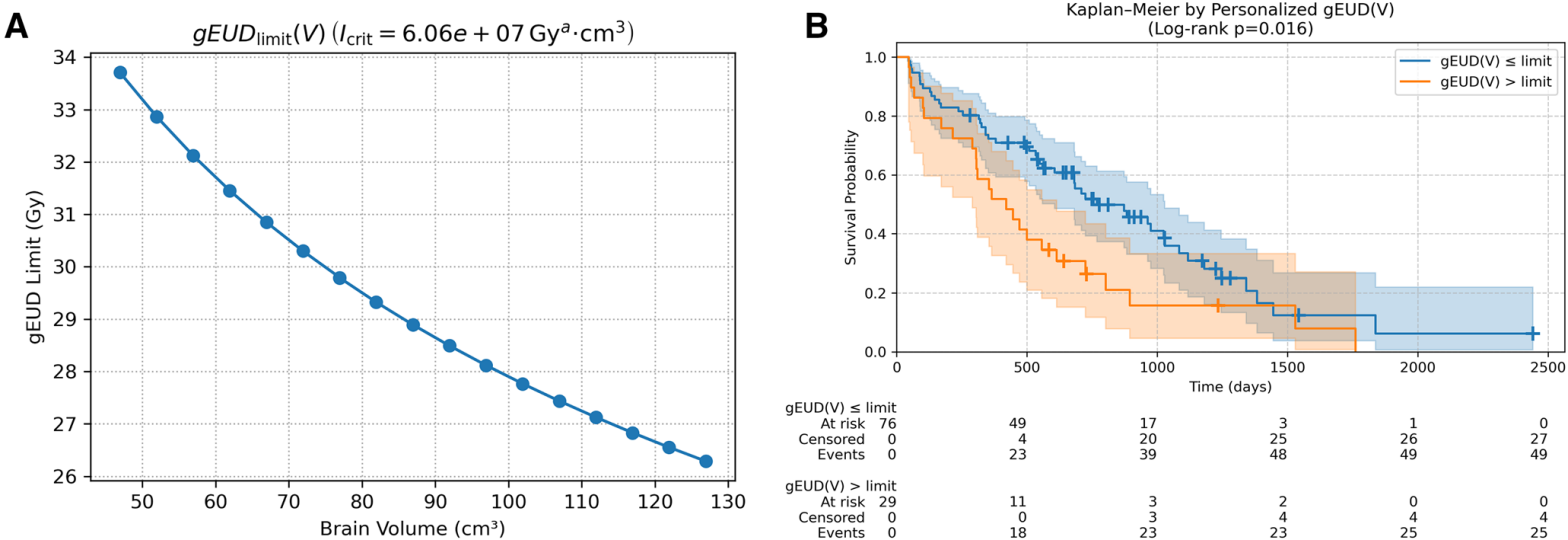
Personalised brain‐dose threshold based on individual brain volume. (A) Relationship between the personalised gEUD_max_(*V*) ceiling and brain volume in the cohort. (B) Kaplan–Meier curves comparing OS for dogs whose whole‐brain gEUD remained under versus exceeded their personalised gEUD ceiling. (Median OS was 769 days for dogs within their limit versus 420 days if exceeded.).

We identified an optimal integral threshold 𝐼_crit_ = 6.06 × 10^7^Gy^4^ cm^3^. This corresponds to a personalised 𝑔𝐸𝑈𝐷_max_(*V*) ceiling that varies with brain volume—for example, ~33 Gy for a 60 cm^3^ brain versus ~29 Gy for a 100 cm^3^ brain. Figure 3A shows the resulting gEUD ceiling across the brain‐volume range of our cohort (47–124 cm^3^). Figure 3B presents Kaplan–Meier curves for dogs whose treatment plans exceeded versus respected their personalised gEUD ceiling (median OS 420 d vs. 769 d; HR = 1.81, 95% CI: 1.11–2.95, *p* = 0.017). Time‐dependent classification performance showed that personalised thresholds improved sensitivity at 1, 2 and 3 years (+8.6, +7.3, +6.2 percentage points, respectively) at the cost of modest specificity losses (−4.3, −2.9, −12.6 points). On average, sensitivity rose by ~7.4 pp and specificity dropped by ~6.6 pp compared to a fixed 29.7 Gy threshold.

Bootstrap stability of the personalised integral threshold was moderate. Across 1000 resamples, the median *I*_crit_ was 4.44 × 10^7^ Gy^4^ cm^3^ (IQR: 3.94 × 10^7^ to 5.28 × 10^7^). The median adjusted HR for exceeding the personalised ceiling was 1.59 (IQR: 0.58–2.96), with 40.5% of resamples reaching adjusted *p* < 0.05 (unadjusted 43.3%). These results indicate a consistent directional effect (HR > 1 on average) with variability in statistical significance under resampling.

### 3.8 Tumour Location and Sensitivity Analysis

Median overall survival for pituitary tumours was 975 days (~32.0 months) and for non‐pituitary tumours was 614 days (~20.2 months); this difference was not statistically significant (log‐rank *p* = 0.239). Because pituitary tumours can be biologically more indolent, we repeated the primary Brain–GTV_V32 analysis after excluding pituitary cases (nonpituitary subset, *n* = 85). The association between Brain–GTV_V32 and survival persisted in this subset: using the pre‐specified threshold of 12, the log‐rank test yielded *p* = 0.009. Median OS in the non‐pituitary subset was 28.7 months for Brain–GTV_V32 ≤ 12 and 16.2 months for Brain–GTV_V32 > 12.

## 4 Discussion

The pooled median overall survival in two randomised trials using a 10 × 4 Gy hypofractionated protocol trials was 681 days (~22.4 months). These outcomes are concordant with other definitive radiotherapy series, which report median overall survival around 18–21 months [1, 2, 3, 4, 5, 6, 7, 15]. By modality and tumour type, stereotactic protocols yield median survival ≈18.5 months in meningioma cohorts (3 × 8 Gy) and ≈12 months in glioma cohorts (mostly 24 Gy in 3 fractions), framing expectations for mixed populations [11, 12, 16]. In pituitary masses, 5‐fraction SRT (30–35 Gy) and 19–20‐fraction definitive RT (50–54 Gy) provide similar survival (overall median ≈20 months) [9].

This analysis demonstrates that dose–volume effects in the brain significantly influence survival in dogs with intracranial tumours. Dogs whose treatment plans limited high‐dose irradiation of normal brain lived longer than those with greater normal brain exposure. Specifically, Brain–GTV volumes exceeding ~10 to 12 cm^3^ at 30–35 Gy were associated with markedly poorer survival, an effect that remained significant after adjusting for tumour size and body weight. These findings suggest that high‐dose radiation to normal brain contributes directly to increased mortality—likely through radiation‐induced normal‐tissue injury (e.g., necrosis with neurologic sequelae)—rather than merely reflecting more aggressive disease or larger tumours.

Bootstrap resampling generally supported the stability of the principal thresholds (e.g., Brain–GTV V32 median bootstrapped cutoff = 11.41 cm^3^, IQR: 11.22–11.50 cm^3^). For the personalised integral ceiling, bootstrap results were more variable: across 1000 resamples the median *I*_crit_ was 4.44 × 10^7^ Gy^4^ cm^3^ (IQR: 3.94 × 10^7^ to 5.28 × 10^7^) and the median adjusted HR for exceeding the personalised ceiling was 1.59 (IQR: 0.58–2.96); 40.5% of resamples produced adjusted *p* < 0.05. These findings indicate consistent directional effect for the personalised ceiling (HR > 1 on average) but also moderate instability of its exact numeric cutoff under resampling. Therefore, the personalised gEUD ceiling is presented as promising and exploratory and should be prospectively validated; in contrast, fixed Brain–GTV V32 thresholds were robust under both FDR correction and bootstrap.

In addition, controlling the false discovery rate within the pre‐specified family of five high‐dose metrics left V32, V35 and V38 significant under both BH and BY procedures, with V29 significant and whole‐brain gEUD significant under BH and marginal under BY, further supporting that these associations are not artefacts of multiple testing.

Based on these results, we propose evidence‐based dose‐volume guidelines for a 10‐fraction canine intracranial radiotherapy schedule. Table 3 summarises the key dose–volume thresholds identified in this study and their associated survival outcomes. These suggested thresholds can serve as planning goals or limits in future veterinary radiotherapy cases.

**TABLE 3:** Suggested dose–volume thresholds for normal canine brain and associated survival impact.

Parameter (structure)	Threshold	Median OS if≤threshold	Median OS if>threshold
Brain–GTV V32 Gy	≤ 13 cm^3^	29.1 month	16.7 month
Brain–GTV V35 Gy	≤ 10 cm^3^	29.4 month	16.7 month
Brain–GTV V38 Gy	≤ 8 cm^3^	29.4 month	16.7 month
Whole‐brain gEUD	≤ 30 Gy	24.1 month	12.2 month

Based on our findings, we propose the following brain dose–volume recommendations for a 10‐fraction (10 × 4 Gy) canine intracranial radiotherapy:

- *Limit intermediate‐dose normal brain volume*: Keep the volume of normal brain receiving ~30 to 35 Gy below roughly 10–13 cm^3^; staying below these limits may significantly improve survival.
- *Minimise overall brain dose*: Aim to keep the whole‐brain gEUD at or below 30 Gy. Exceeding these thresholds corresponded to ~1.7 to 2.3‐fold higher mortality hazard in this cohort.

Incorporating these normal‐brain constraints alongside established tumour coverage objectives enables a balanced approach that preserves neurological function while effectively treating the tumour. By applying these quantitative thresholds, clinicians can potentially improve patient longevity and quality of life, based on evidence‐based guidance tailored to veterinary neuro‐oncology.**

Our canine dose–volume thresholds align with principles established in human neuro‐oncology, although direct extrapolation warrants caution. In human single‐fraction stereotactic radiosurgery, limiting the volume of normal brain receiving 12 Gy (V12) to approximately 5–10 cm^3^ is an accepted guideline to reduce radionecrosis risk [17]. Biological estimation using Linear‐Quadratic model, with *α*/*β* = 2 Gy, provides that ~32 Gy in 10 fractions (as used in our canine trials) equates to a single‐fraction dose of ~12 Gy (EQD2/2≈42Gy). Likewise, human 3‐fraction data recommend V18 Gy < 30 cm^3^ and V23 Gy < 7 cm^3^ to keep radionecrosis risk < 10%. In our study, dogs with normal‐brain volumes > 12 cm^3^ at 32 Gy had significantly worse survival, mirroring the volume–toxicity relationships observed in humans. These cross‐species parallels highlight dose‐volume relationships that critically influence normal‐brain tolerance and outcomes.

Leveraging prospective data from two randomised trials, this study provides a comprehensive evaluation of normal‐brain dose–volume effects in canine radiotherapy. The inclusion of both uniform and heterogeneous (dose‐escalation) protocols enhances the generalisability of the findings, indicating that the observed dose–volume relationships reflect fundamental normal‐tissue responses rather than trial‐specific planning techniques. We surveyed 44 dose–volume parameters and applied an objective, automated cutoff‐finding algorithm, followed by Cox modelling and residual adjustment for tumour volume and body weight, to derive and validate clinically relevant cut‐points.

Despite these strengths, several limitations merit consideration. First, although 105 dogs represent a substantial veterinary cohort, the sample size remains moderate and limits statistical power for some subgroup analyses. Second, the exploratory testing of numerous metrics raises the possibility of false positives due to multiple comparisons. Future work should include statistical correction methods or independent validation to confirm these thresholds. Third, pooling two trials introduces some heterogeneity in techniques and patient populations; unmeasured factors (e.g., imaging or contouring differences) may have influenced results. However, the consistency of dose–volume effects across the different protocols supports the robustness of our findings. Fourth, using OS as the endpoint in veterinary patients may be confounded by owner‐driven euthanasia decisions. While clinical observations implicate treatment‐related neurologic decline in some cases, we did not directly consider neurologic function or tumour progression in this study—future studies should incorporate such endpoints or quality‐of‐life measures. Finally, our recommendations are specific to a 10‐fraction regimen (40–48 Gy total) and may not directly apply to more protracted or single‐fraction schedules. For example, in a conventional 2 Gy per fraction regimen, the equivalent dose thresholds would be higher and larger brain volumes might be tolerated. Therefore, caution is warranted when extrapolating beyond the studied fractionation schemes.

## 5 Conclusion

Our analysis demonstrates that specific brain dose‐volume parameters have a profound impact on survival in dogs undergoing 10‐fraction radiotherapy for intracranial tumours. Limiting Brain–GTV volumes to ~10 to 12 cm^3^ at 30–35 Gy and keeping whole‐brain gEUD ≤ 30 Gy (or within personalised limits based on brain volume) were associated with significantly prolonged survival. These evidence‐based guidelines fill a critical gap in veterinary neuro‐oncology by providing quantitative, clinically actionable dose constraints. Applying these thresholds alongside other organ‐at‐risk limits and tumour coverage objectives enables clinicians to optimise treatment plans for both efficacy and safety, ultimately improving patient longevity and quality of life.

## Funding

The two trials included in this work were supported by the Swiss National Science Foundation (SNSF) (320030–182490); Principal Investigator: Carla Rohrer Bley.

## Conflicts of Interest

The authors declare no conflicts of interest.

## Supporting information


**Table S1:** Dose–volume thresholds associated with overall survival (OS).

## Data Availability

The data that support the findings of this study are available from the corresponding author upon reasonable request.
